# Diagnostic Accuracy of Digitized Conventional Radiographs by Camera and Scanner in Detection of Proximal Caries

**DOI:** 10.5681/joddd.2009.031

**Published:** 2009-12-15

**Authors:** Solmaz Valizadeh, Mohammad Amin Tavakoli, Tara Zarabian, Farzad Esmaeili

**Affiliations:** ^1^ Assistant Professor, Department of Oral and Maxillofacial Radiology, Shahid Beheshti University of Medical Sciences, Tehran, Iran; ^2^ Professor, Department of Oral and Maxillofacial Radiology, Faculty of Dentistry, Shahid Beheshti University of Medical Sciences, Tehran, Iran; ^3^ Post-graduate student, Department of Pediatrics, Faculty of Dentistry, Azad University of Medical Sciences, Tehran, Iran; ^4^ Assistant Professor, Department of Oral and Maxillofacial Radiology, Faculty of Dentistry, Tabriz University of Medical Sciences, Tabriz, Iran

**Keywords:** Proximal caries, conventional radiographs, digital camera, scanner

## Abstract

**Background and aims:**

Digital radiographs have some advantages over conventional ones. Application of digital recep-tors is not routine yet. Therefore, there is a need for digitizing conventional radiographs. The aim of the present study was to compare the diagnostic accuracy of digitized conventional radiographs by scanner and camera in detection of proximal car-ies.

**Materials and methods:**

Three hundred and sixteen surfaces of 158 extracted posterior teeth were radiographed. The radiographs were digitized using a digital camera and a scanner. Five observers scored the images for the presence and depth of caries. Histopathologic sections were the gold standard. Kappa agreement coefficient was used for statistical analysis.

**Results:**

Kappa agreement coefficients between the camera and the scanner and also between each one with the gold stan-dard in detecting the depth of caries were 0.504, 0.557 and 0.454, respectively. In detection of caries, the indexes were 0.571, 0.553 and 0.527, respectively.

**Conclusion:**

Diagnostic accuracy of camera images in caries detection was more than that of scanned images, but there was also a moderate consistency between them. The consistency of detecting the presence of caries was more than that of detecting their depths. It seems that both digital cameras and scanners can be used interchangeably.

## Introduction

Outstanding progresses in computer technology have had a great influence on dentistry and dental radiography field, including digital imaging systems. Digital images are prepared in two ways: 1) by the use of digital receptors instead of films, in which images can be displayed directly on a computer; 2) use of systems for digitizing analog images.^1^ Application of digital receptors is not routine yet and conventional radiographs are being used in most cases. Thus, until digital radiographic systems gain a firm foothold, there is a need for digitizing conventional radiographs in order to display them on a computer.^2^

The use of digital images instead of conventional radiographs has many advantages, such as the ability to enhance digital images using computer software, image compression in order to save them in a smaller size, more proper saving of patients’ files, preparing educational files for students in educational centers and rapid transmission of digital information of patients to other centers for counseling.^1 , 3^ Utilizable digitizers are scanners and digital cameras which allow the images to be displayed digitally on a computer by altering the analog nature of films’ information to digital. Each of these systems has some advantages and disadvantages. A scanner is a steady motionless apparatus but accurate ones are relatively expensive. Cameras are readily available and produce nearly real-time digital images but there would be the risk of movement and vibration during picture taking. The mechanisms of picture production in these two systems are different.^2^

A large number of studies have assessed the quality of radiographic images digitized by digital cameras and scanners at various resolutions. The result of assessing the quality of images at various resolutions of the camera has shown that the use of the highest possible camera resolution in order to achieve more diagnostic accuracy is not necassary.^1^ The study of various resolutions of the scanner has yielded the same results.^2^ These studies have not compared these two systems. There was just one similar study in which high interference in the final diagnosis was inevitable because of the large number of observers in the study.^4^ Regarding the fact that radiographs are extensively used in dental caries detection and usually only one of these digitizers (camera or scanner) is available, we decided to compare the diagnostic accuracy of scanner and camera digitizers in detection of proximal caries.

## Materials and methods

In this experimental in vitro study, according to the literature and epidemiologic studies,^4^ 158 human posterior teeth, extracted for different reasons were prepared by random sampling method. Because all the proximal surfaces were assessed, the overall number of the samples was 316. The teeth with visible cavities, restorations, abrasions, fractures or dental anomalies were excluded. The teeth were sterilized in 10% formalin for 24 hours; then each set of three teeth were placed in contact in blocks of plaster and sawdust and a number was given to each block. Each block was exposed by the x-ray machine Gendex 765 DC (Des Plaines, IL, USA) at 65 kVp, 7 mA, and the time recommended for E-speed films. A device was made for stabilizing the blocks and the collimator and maintaining the tube-film distance (25 cm), and an 18 mm plexy glass slab was used to simulate soft tissues. Reproducible projections could be prepared with this device. The exposed E-films were developed by an automatic film processor (Gendex, Clarimat 300, London, UK). In the first step, digital images of conventional radiographs were taken by a digital camera (Canon IXY, Canon Inc, Tokyo, Japan, 10 Megapixels) at 1280 × 960 resolution. The distance between the camera's lens and radiographs was kept at 5 cm by making a stabilizing device. In the following step all the conventional radiographs were digitized by scanner (Mikrotek Scan Maker 6900 XL, Taiwan, R.O.C) at 300 dpi.

The obtained digital images were saved by the Photoshop CS2 9.02 software.

Five observers, three radiologists and two general practitioners, were asked to assess the images regarding the presence and the depth of caries. Before image evaluation, the observers were instructed in using the software and the enhancing facilities of images, including the change of contrast, density and magnification. In order to reduce the differences between the diagnostic quality of general practitioners and radiologists, the dentists were instructed by a radiologist. The viewing conditions of the observers were similar and they assessed the images in two steps (one for the camera and one for the scanner). To eliminate the memory effect, they were given one week’s time out between the two stages, and the observers were not informed about the number of each block. The presence and the depth of proximal caries were numerated in the following manner: 0 = no caries, 1 = enamel caries, 2 = DEJ or dentin caries.

Histopathologic sections were used as the gold standard to validate the above-mentioned variables. To this end, the teeth were mounted in translucent acryl and were sectioned by diamond discs with 0.15 nm thickness in mesiodistal direction, from the height of contour and under the pathologist's supervision. The sections were then observed under a stereoscope (Olympus SZX9, 1×2, Tokyo, Japan) from both directions and were confirmed by the pathologist. Demineralization was observed in the form of white-opaque to dark brown discoloration in carious areas.

In order to assess the diagnostic accuracy of each digitizer system in detecting the presence and the depth of caries and to assess the diagnostic accuracy of these systems and comparing them with histopathologic findings as the gold standard, Kappa coefficient was used. Inter-observer reliability was measured by Cronbach’s Alpha.

**Table 1:** Diagnostic test performance

	Digitizer	
Indicator	Camera	Scanner
Specificity	97	92.8
False positive	2.9	7.1
Negative indicative value	85.9	86.7
Sensitivity	50.6	55.8
False negative	49.3	44.1
Positive indicative value	84.7	71.6

## Results

**Figure 1:**
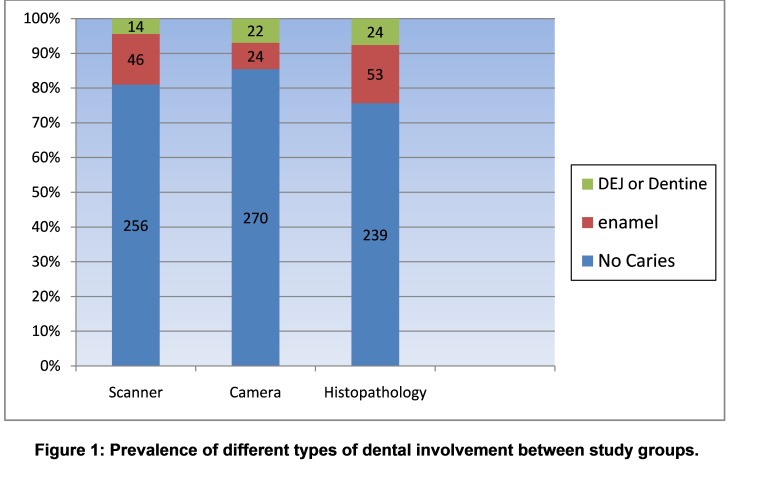
Prevalence of different types of dental involvement in the study groups.

Cronbach’s Alpha index was 0.832, which indicates that the observers' viewpoints were very close to each other; therefore, to continue the calculation their average scores given to the images were used. Figure 1 shows the prevalence of different types of dental involvement in the study groups.

The measures of agreement (Kappa) with the gold standard in detecting the depth of caries were 0.557 and 0.454 for the camera and the scanner, respectively. According to the higher measure of agreement for the camera, camera images were found to correlate better with the histological caries depth in proximal surfaces.

Kappa agreement coefficient was applied to evaluate whether the differences in diagnostic accuracy between the camera and the scanner in detecting the depth of caries was significant or not.

The obtained number of 0.504 shows a moderate agreement between the camera and scanner images in diagnosing the depth of proximal caries.

The measure of agreement with the gold standard in detecting the presence of caries was 0.553 for the camera and 0.527 for the scanner.

These results indicate that the diagnostic accuracy of both series of camera and scanner images in determining the presence of caries compared with the gold standard was relatively reliable.

The indicators relating to the accuracy of diagnostic performance in the camera and scanner (Table 1) and the Receiver Operating Characteristic (ROC) curve were drawn (Figure 2).

Figure 2. ROC curves of camera and scanner.

**Scanner:**
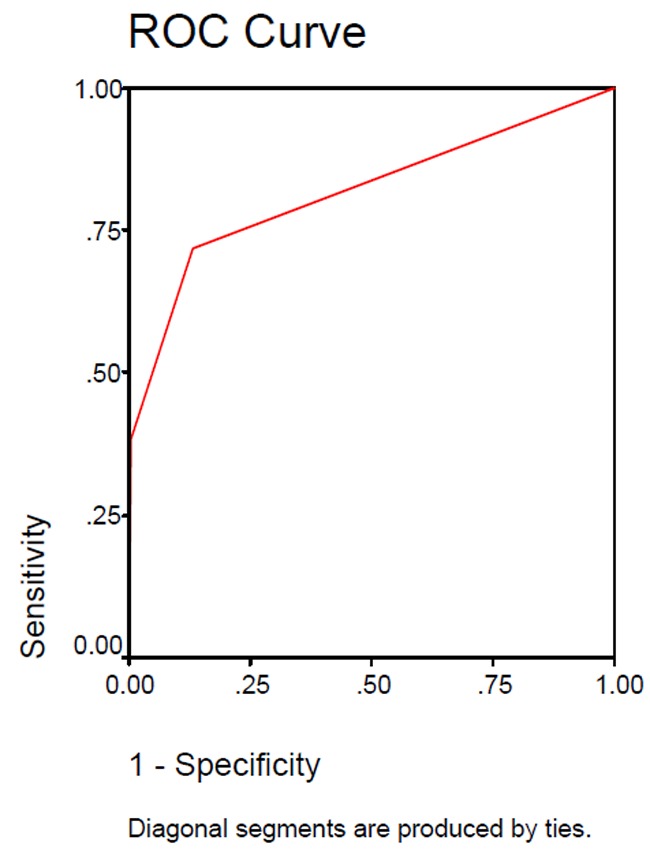

**Camera:**
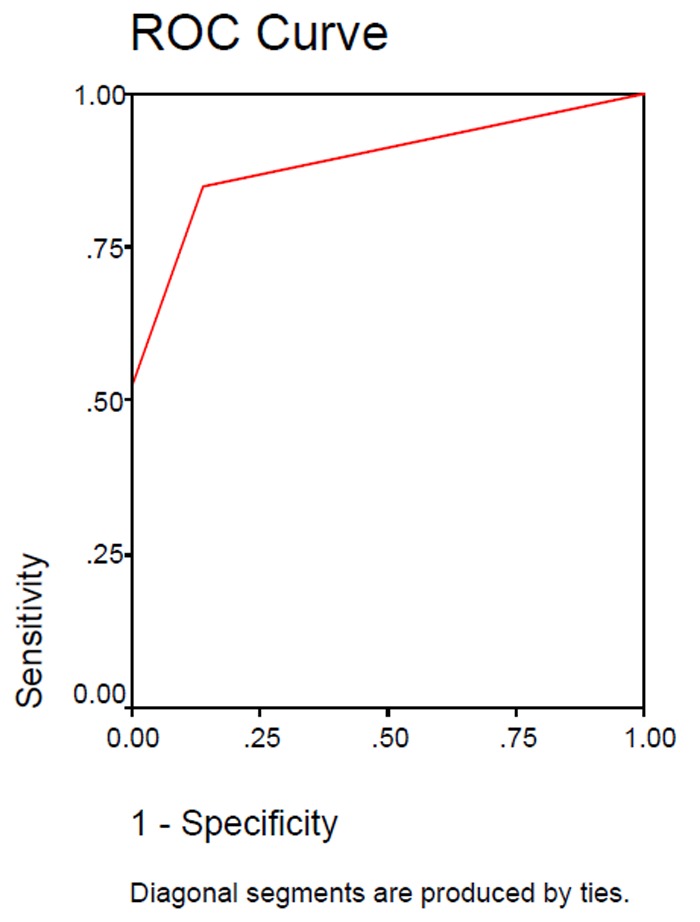

In a ROC curve, the vertical line indicates sensitivity and the horizontal one shows specificity. The assessment is made by measuring the area under the curve. The closer the obtained number is to one, the better the sensitivity and the specificity. The area under the ROC curve for the camera and the scanner were 0.890 and 0.816, respectively. Thus, both series of camera and scanner images are good enough regarding sensitivity and specificity.

Kappa agreement coefficient was used to compare the diagnostic accuracy of camera and scanner images in detecting the presence of caries. The measure of agreement between the camera and the scanner in detecting the presence of caries was 0.571, which shows a moderate agreement between the two sets of images in detecting the presence of caries.

Apparently, the measure of agreement between camera and scanner images in diagnosing the presence of caries is higher than detecting the caries depth.

**Figure 3.**
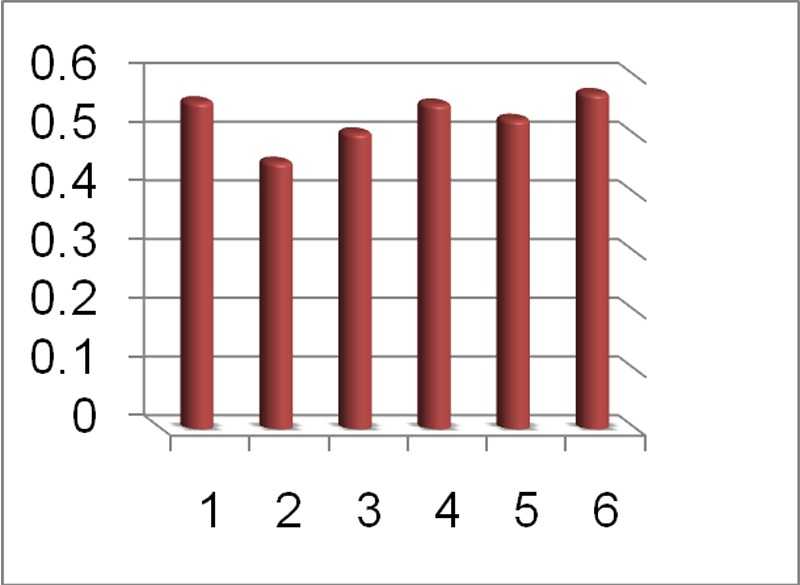
Measures of agreement between camera, scanner and the gold standard in detecting the depth and the presence of caries.

Higher Kappa value for the camera indicates that the diagnostic accuracy of images captured by the digital camera in detecting the depth of caries is slightly better than that of the scanner, compared with the gold standard. But by studying the measure of agreement in caries depth determination, a moderate agreement is observed between them. All of the measures of agreement are shown in Figure 3.

1. Measure of agreement between the camera and the gold standard in detecting the presence of caries.

2. Measure of agreement between the scanner and the gold standard in detecting the presence of caries.

3. Measure of agreement between the camera and the scanner in detecting the presence of caries.

4. Measure of agreement between the camera and the gold standard in detecting the depth of caries.

5. Measure of agreement between the scanner and the gold standard in detecting the depth of caries.

6. Measure of agreement between the camera and the scanner in detecting the depth of caries.

The results of this study indicate that both sets of images obtained by the camera and the scanner do well in detecting the presence of caries, compared with the gold standard and Kappa coefficient shows a moderate agreement in detecting the presence of caries between them.

## Discussion

Radiography along with clinical examination is the most important tool in caries diagnosis.^5^ There are two imaging systems: analog and digital. Although use of digital radiographs with receptors, like CCD and PSP, is widely accepted for dental clinical applications, they are not routine yet. Therefore a combination system, in which conventional radiographs are digitized, is still needed. Digital cameras or scanners can be used to digitize conventional images.

Multiple studies have compared the diagnostic accuracy of conventional radiographs with the digitized ones in detecting proximal caries.^6 - 8^

In this study the diagnostic accuracy of digitized radiographs by two different digitizers (camera and scanner) in detecting the proximal caries was compared.

In order to obtain digital images from conventional radiographs with the camera, we used the 1280×960 resolution. According to Prapayasatok et al, when there is appropriate resolution, higher resolution will not improve the quality.^1^

In order to obtain digital images of conventional radiographs with the scanner, the resolution of 300 dpi was used. According to the Janhom et al, this resolution is the best resolution for conventional radiograph scans and the obtained images by higher resolutions do not have an improved diagnostic accuracy.^2^

None of the digital images were compressed, although Pabla et al^9^ have reported that a specific range of lossy compress does not greatly affect determination of proximal caries, but before these techniques can be used routinely, more studies in this field are needed.

In this study, there was not much concern about choosing the monitor on which the images were to be displayed because Cederberg et al^10^ have shown that displaying digital images on different monitors does not affect their quality.

The observers were permitted to use the enhancement facilities because the enhancement of an image such as altering contrast, density and magnification, may improve the quality of the displayed image by altering the digital information. In caries detection, enhanced images compared with the original ones have significantly improved the diagnostic accuracy.^11^

In some studies, determining the presence of caries has been conducted on a 5-point scale: 1 = definitely no caries; 2 = probably no caries; 3 = uncertain; 4 = probably with caries; and 5 = definite caries.^1^ The problem with this kind of scale is that besides the subjectivizing of variables, the depth of caries will not be located. In this study in order to have a more accurate assessment as well as to determine the depth of caries, a 3-point scale was used: 0 = no caries; 1 = enamel caries; and 2 = DEJ or dentin caries.^12^

Numerous studies have shown significant differences in the diagnostic accuracy by different observers.^12^ This difference might be attributed to differences in experience, education or visual conception of the observers.^13^ According to previous studies, general practitioners might have less diagnostic accuracy than specialists, whereas radiologists, regardless of the imaging modality, do quite better than the general practitioners and determine the depth of caries more accurately. In this study, Cronbach’s Alpha was 0.832, which indicates that the observers’ scores were very close to each other, which might be attributed to educating the non-specialists prior to observing and instructing them in how to assess the images.

According to the measure of agreement (Kappa) between the digital camera and the gold standard and also the scanner and the gold standard, when only the presence of caries is assessed, the diagnostic accuracy of both sets of images is higher than the situation in which the depth of caries is assessed. Moreover, the diagnostic test performance indicated that both camera and scanner images estimated the number of sound surfaces less than the real quantity and the false negative was 49.3% for the camera and 44.1% for the scanner. Both series of camera and scanner images in studies carried out by Khan et al^11^ and Wenzel et al^12^ estimated the number of carious lesions less than the actual quantity.

Sensitivities for the camera and the scanner were 50.6% and 55.8%, respectively. These results are logical because studies and lots of sources have demonstrated that most initial carious lesions cannot be visualized on radiographs until they penetrate into half of the enamel thickness. Since the posterior teeth have often broader proximal surfaces, detecting a small amount of demineralization and the progressive zone of active carious lesions in radiographs is difficult.^1^

Lesions limited to enamel might not be seen on radiographs until there has been 30-40% of demineralization; therefore, the actual depth of caries is often much more than what is seen on radiographs and radiographic images show caries depth less than their actual depth. For instance, sensitivity of bitewing radiographs in caries detection is estimated to be only 40-65%. Cases in which the demineralization cannot yet be seen on the radiograph are considered false negative. About half of proximal lesions in enamel cannot be detected by radiographs; therefore, they cannot be detected on these digital images, either.^1^

False positive rate for the scanner was 7.9%, which indicates that more accuracy is needed while assessing scanner images. The results of the present study demonstrated that the diagnostic accuracy of both series of digital images obtained by the camera and the scanner was comparable in detecting the presence and the depth of caries with no significant differences between them. The measure of agreement between the camera and the scanner indicates that in detecting the presence and depth of caries, there is a moderate agreement and the agreement about determining the presence of caries is higher. These results coincide with the results of a previous study,^4^ in which the diagnostic quality of the scanner and the commercial digital images was similar.

## Conclusion

We can conclude from the results of the present study that digital cameras and scanners could replace each other for digitizing conventional radiographs. Digital cameras are more commonly usage, have less volume and weight, are portable, their application is quite popular, do not need special instructions and are generally less expensive; therefore, using digital cameras for digitizing conventional radiographs in order to assess proximal caries can be suggested.
